# Lysine Methyltransferase NSD1 and Cancers: Any Role in Melanoma?

**DOI:** 10.3390/cancers14194865

**Published:** 2022-10-05

**Authors:** Imène Krossa, Thomas Strub, Andrew E. Aplin, Robert Ballotti, Corine Bertolotto

**Affiliations:** 1 Université Côte d’Azur, 06100 Nice, France; 2 Team 1, Biology and Pathologies of melanocytes, Inserm, Equipe labellisée Ligue 2020 and Equipe labellisée ARC 2022, Centre Méditerranéen de Médecine Moléculaire, 06200 Nice, France; 3 Department of Pharmacology, Physiology, and Cancer Biology, Thomas Jefferson University, Philadelphia, PA 19107, USA

**Keywords:** epigenetics, cancers, melanoma, therapies

## Abstract

**Simple Summary:**

Epigenetic events, which comprise post-translational modifications of histone tails or DNA methylation, control gene expression by altering chromatin structure without change in the DNA sequence. Histone tails modifications are driven by specific cellular enzymes such as histone methyltransferases or histone acetylases, which play a key role in regulating diverse biological processes. Their alteration may have consequences on growth and tumorigenesis.

**Abstract:**

Epigenetic regulations, that comprise histone modifications and DNA methylation, are essential to processes as diverse as development and cancer. Among the histone post-translational modifications, lysine methylation represents one of the most important dynamic marks. Here, we focused on methyltransferases of the nuclear binding SET domain 1 (NSD) family, that catalyze the mono- and di-methylation of histone H3 lysine 36. We review the loss of function mutations of *NSD1* in humans that are the main cause of SOTOS syndrome, a disease associated with an increased risk of developing cancer. We then report the role of NSD1 in triggering tumor suppressive or promoter functions according to the tissue context and we discuss the role of NSD1 in melanoma. Finally, we examine the ongoing efforts to target NSD1 signaling in cancers.

## 1. Introduction

Epigenetic events serve to remodel chromatin structure without nucleotide change, which subsequently lead to gene regulation and expression. They comprise post-translational modifications of histone tails, DNA methylation, nucleosome occupancy, non-coding RNA regulation and RNA editing [1]. Post-translational modifications of histone tails are driven by specific cellular enzymes such as histone methyltransferases or histone acetylases which determine the status of histone methylation or acetylation, respectively [2].

Among the histone post-translational modifications, lysine methylation represents one of the most important dynamic marks. Histone lysine methylation is deposited, recognized and removed by a set of specific lysine methyltransferases, effector proteins, and demethylases, respectively, whose expression depends on the cellular context [3]. Their downstream biological effects are mediated by methyllysine-binding proteins, and they are associated with either active transcription (such as H3K4me or H3K36me2) or repressed transcription (such as H3K27me or H3K9me) [4,5].

As such, these modifications play a key role in regulating diverse biological processes such as cell cycle, DNA repair and genomic integrity maintenance amongst a multitude of other processes.

In this review, we discuss the role of the Nuclear binding SET Domain 1 (NSD) family of histone H3 lysine 36 methyltransferases in developmental disorders and cancer. This family comprises *NSD1* (nuclear receptor SET (su(var)3–9, enhancer-of-zeste, trithorax) domain containing protein-1/KMT3B) and its two homologs, *NSD2* (WHSC1) and *NSD3* (WHSC1L1) [6].

## 2. NSD1 Structure and Function

### 2.1. Physiological Function

NSD proteins share a highly conserved catalytic SET domain, further subdivided into pre-SET, SET and Post-SET domains. They exhibit additional domains, such as multiple Proline-Tryptophan-Tryptophan-Proline (PWWP) and Plant Homeodomain (PHD) finger regions, which are responsible for chromatin and protein interactions [7,8,9]. Multiple putative nuclear localization signal (NLS) domains have been also detected, which allow the translocation of NSD proteins to the nucleus [10,11].

**Figure 1:**
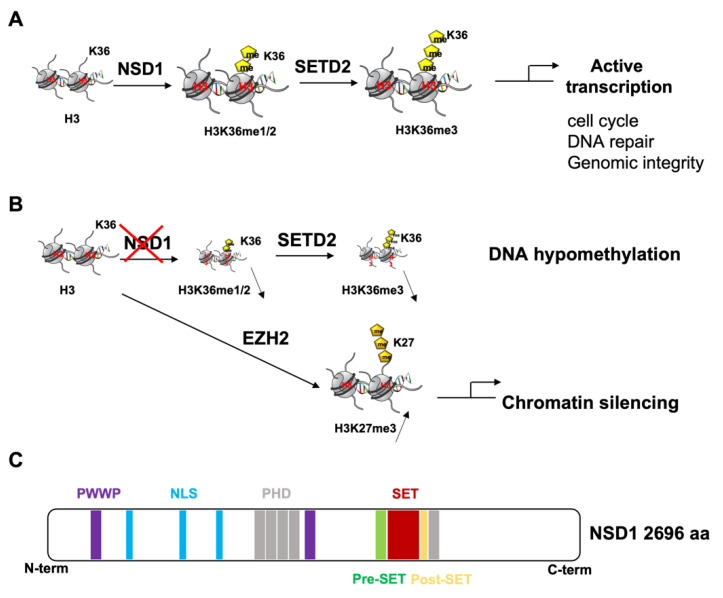
Epigenetic mechanisms driven by the histone lysine methyltransferases NSD1 and SETD2. (**A**) NSD1 and SETD2 regulate cell cycle, DNA repair and genomic integrity. (**B**) NSD1 loss reduces H3K36me2 active transcriptional methylation marks, leading on one hand to H3K36me3 decrease known to be involved in DNA hypomethylation, and on the other hand causing a genome wide accumulation of H3K27me3, a repressive histone modification associated with chromatin silencing, mediated by PRC2-catalytic subunit Enhancer of zeste homolog 2 (EZH2). (**C**) Schematic representation of NSD1 architecture showing the different protein domains. PWWP: proline–tryptophan–tryptophan–proline). NLS: nuclear translocation signals. PHD plant homeodomain zinc fingers. SET: Su(var)3-9, Enhancer-of-zeste, Trithorax.

NSD proteins catalyze the mono- and di-methylation of H3K36, which then serve as substrates for trimethylation by SETD2 [12,13] (Figure 1A).

Methylation of H3K36 is found in species ranging from yeasts to mammals. It plays a crucial role in regulating gene transcription activation, maintaining genomic integrity and stability, DNA repair, cell cycle and even nutrient stress response [3,14,15] (Figure 1A).

Human *NSD1* gene, located on chromosome 5q35, encodes two protein isoforms of 2696 aa and 2427 aa. Lucio-Eterovic and coworkers have demonstrated that the short NSD1 isoform is predominantly expressed in a variety of cell types including fetal/adult brain, kidney, skeletal muscle, spleen and the thymus whereas the long isoform is less abundant [16,17]. *NSD1* heterozygous mutant mouse embryo is viable and fertile but *NSD1* knockout is lethal, demonstrating its importance in embryonic development [18].

NSD1 has the ability to bind near various promoter regions and interact with H3K36 and RNA polymerase II (RNAPII) to regulate transcription and promote gene expression by stimulating the RNAPII-mediated elongation [16]. Through binding to various methylated states of histone H3 via its PHD domains, and to cofactors, NSD1 behaves like a cofactor complex to target regulatory DNA. Consequently, NSD1 effect will depend on the cellular context, methylated states of these histones, and activities of cofactors that it recruits.

NSD1′s loss leads to DNA hypomethylation, highlighting a possible interplay between epigenetic histone methylation marks and DNA methylation [19] (Figure 1B). In line with that, Choufani and collaborators showed that *NSD1′s* haploinsufficiency triggers a specific genome-wide pattern of DNA methylation alterations, which might represent a highly sensitive and robust diagnostic tool for Sotos syndrome compared to other overgrowth development syndromes [20]. It has been postulated that *NSD1′s* disruption affecting H3K36 methylation could, in turn, prevent these histones from recruiting DNA methyltransferases [21,22].

When altered, the various *NSD1* functions mentioned above contribute to the pathogenesis of a variety of human congenital developmental syndromes and cancerous diseases which shall be discussed further in this review.

### 2.2. NSD1′s Role in Congenital Developmental Disorders

*NSD1* represents one of the key regulators of development. In humans, germline alterations (including missense, truncating and splice-site mutations and submicroscopic deletions) of *NSD1* potentially inducing loss-of-function of the NSD1 protein have been associated with a developmental syndrome called Sotos. Sotos is characterized by a distinctive facial appearance, physical overgrowth, advanced bone age, learning disabilities and cancer predisposition [9,23,24,25].

Kurotaki and coworkers established that 77% of individuals diagnosed with the Sotos syndrome are genetically characterized with microdeletions or point mutations impacting the entire *NSD1* gene [25]. A total of 87 *NSD1* point mutations and 58 microdeletions have been reported, and the frequencies of each differ among populations. For instance, the Japanese population harbors microdeletions in the majority of cases, whereas in European populations, *NSD1* intragenic inactivating mutations account for the majority of Sotos cases [26,27]. A strong correlation between presence of an *NSD1* alteration and clinical phenotype was reported [28]. In the Sotos syndrome, the truncating mutations were spread throughout *NSD1,* but there was evidence of clustering of missense mutations in highly conserved functional domains. Likewise, the position of mutations in the patients with Weaver syndrome has been described to reflect a genotype-phenotype correlation [28].

Moreover, why there are different rearrangement frequencies among the populations studied remain to be determined.

Although *NSD1′s* alterations have primarily been incriminated in the overgrowth Sotos syndrome, additional NSD1 disruption have been depicted in other developmental syndromes. Indeed, *NSD1* mutations have also been observed in unexplained cases of a congenital overgrowth syndrome known as Beckwith-Wiedemann syndrome (BWS), which is characterized by macroglossia, visceromegaly, umbilical hernia, abdominal wall defect and a predisposition to cancer. It is worth noting that microduplications of 5q35.2–q35.3 comprising the *NSD1* gene locus have been described in rare patients with a clinically reversed Sotos syndrome. These individuals are characterized by short stature, microcephaly, learning or mild to moderate intellectual disabilities, and distinctive facial features. *NSD1* 5q35 duplication has been also detected in patients with Silver-Russell syndrome (SRS) features, which is a growth retardation disorder characterized by facial dysmorphia and body asymmetry [29]. These observations suggest that the NSD1 gene dosage settles the phenotype of these developmental syndromes [25,30].

To understand the mechanisms underlying the Sotos syndrome, Brennan and co-workers studied the transcriptomic and DNA methylation profiles in Sotos syndrome patients and healthy control individuals. Their results indicate that NSD1-deposited H3K36 methylation regulates transcription by directing promoter DNA methylation, partially by repressing polycomb repressive complex 2 (PRC2) activity [31].

Bearing in mind that normally NSD1 binds to both transcription co-factors and methylated histones to regulate gene expression, Kurotaki et al.’s findings suggest that NSD1 could also repress gene expression [25,26,30]. Heterozygous inactivation of *NSD1* may result in repression loss of growth promoting genes [18]. The mechanisms by which NSD1 functions as a gene expression repressor is largely unknown, yet it has been shown in Nsd1-knockout mouse embryonic stem cells that H3K27ac increases in parallel with H3K36me2 decrease at active enhancers. Briefly, Nsd1 deposits H3K36me2 and recruits HDAC1 at active enhancers to serve as a ‘safeguard’, preventing further activation of active enhancer-associated genes [32].

*NSD1* loss, which translates into reduced H3K36me2 active transcriptional methylation marks, has also been shown to cause a genome wide accumulation of H3K27me3, a repressive histone modification associated with chromatin silencing, mediated by PRC2-catalytic subunit Enhancer of zeste homolog 2 (EZH2) [33] (Figure 1B). This implies that on the same histone tail, H3K27me3 is mutually exclusive with the methylation of H3K36 [34,35]. Furthermore, histone modification and DNA methylation can influence each other during development. Actually, histone modifications are fairly transient regulatory marks that are replaced in the longer term by more stable epigenetic mark DNA methylation [36]. As a proof of principle of the interaction between loss of function in H3K36 methylation and DNA methylation status, a specific genome-wide pattern of DNA hypomethylation associated with *NSD1* loss-of-function mutations in Sotos syndrome patients was observed [20]. The signature identified distinguishes pathogenic *NSD1* mutations from control subjects, as well as from cases with the clinically overlapping Weaver syndrome ensuing mutations in the histone methyltransferase *EZH2*.

Recently, Martin-Herranz and coworkers examined the epigenetic clocks, which represent biomarkers for the aging process, in patients with a variety of developmental disorders harboring mutations in proteins of the epigenetic machinery. Using DNA methylation data generated from their blood compared to healthy individuals, the authors demonstrated that loss-of-function mutations in NSD1, which cause Sotos syndrome, substantially accelerate epigenetic aging [37,38].

Their findings firstly revealed that patients with Sotos syndrome showed strong evidence of accelerated epigenetic aging when compared with healthy individuals, which makes their epigenome look a decade older than expected [37]. Secondly, they showed a hypermethylation trend in promoters that are bound by Polycomb group proteins (PRC), which correlated with a high number of cell divisions in tissues and therefore could explain the higher cancer predisposition found in these patients and might as well relate to their overgrowth [37,39]. All of these data imply that there’s a strong correlation between the overgrowth phenotypic features observed in Sotos patients with their high epigenetic acceleration age. The same group also suggested that H3K36 methylation process, which is lost in Sotos patients, could be a key component of the epigenetic maintenance system in humans [37].

One of NSD1′s potential downstream effectors in Sotos syndrome is Adenomatous polyposis coli 2 (*APC2*), a tumor suppressor gene involved in the negative regulation of the Wnt/β-catenin signaling pathway [40,41].

Interestingly, a homozygous loss-of-function mutation in *APC2* has been identified in two siblings with Sotos-like symptoms. Furthermore, *Apc2* knockout mice showed some phenotypic similarities to human Sotos syndrome, i.e., impaired learning and memory abilities, therefore there is a possibility that *APC2* expression is repressed in patients with Sotos syndrome and could affect neuronal functions. In line with this, NSD1 knockdown led to decreased APC2 protein levels, which confirmed that APC2 operates downstream of NSD1 [40,41]. It would be interesting to determine whether *Apc2*-knockout mice would represent a model to study Sotos syndrome.

Later on, Visser and collaborators performed a comprehensive study on dermal fibroblasts from patients with Sotos syndrome to decipher the molecular mechanisms of NSD1 functions [42]. They found a significant association of NSD1 expression with the MAPK signaling pathway, which plays a critical role in cell proliferation and survival. Surprisingly, a reduced MAPK/ERK pathway activity was observed in dermal fibroblasts from Sotos patients.

In conclusion, NSD1 disruption is associated with activation of signaling pathways such as WNT/β-catenin and MAPK that have recognized role in controlling cell survival and proliferation. How these different signaling pathways interact to regulate the excessive proliferation characteristic of the overgrowth Sotos syndrome remains to be determined. It is worth noting that Sotos overgrowth syndrome is very likely cancer-predisposing [43]. Patients with Sotos syndrome under fifteen years old are at an estimated 150% increased risk of developing a malignancy when compared to their healthy counterparts [44]. Sotos syndrome seems not to be related to a specific tumor type [45], being associated with various malignancies including Wilms’s tumor, neuroblastoma, hepatocellular carcinoma, non-Hodgkin’s lymphoma and acute lymphoblastic leukemia [46,47] In cancer cells, methylation within regulatory elements serves to turn off the expression of critical genes, such as tumor suppressor or differentiation genes [48]. Hence, transcriptional silencing of critical genes, resulting from *NSD1* alterations or from other epigenetic regulators translating an aberrant imbalance between the H3K36 and H3K27 transcriptional marks, may have critical implication in tumorigenesis and other diseases.

Therefore, findings in Sotos overgrowth syndrome may serve to better understand NSD1 role in cancer.

### 2.3. NSD1′s Role in Cancer

Epigenetic-based mechanisms leading to carcinogenesis can be partitioned into at least two categories: (1) repression of genes normally active such as tumor suppressor genes, (2) activation of genes normally silenced such as oncogenes. The emergence of these mechanisms is conducted either by a single or a team effort of a variety of histone modifying enzymes [49]. Among these enzymes, lysine histone methyltransferases such as NSD1, EZH2 and SETD2 represent key players in epigenetic regulation and their roles usually intertwine depending on the cellular context. The study of each of these enzymes and their interplay has emerged as a subject of interest in many cancers.

**Figure 2:**
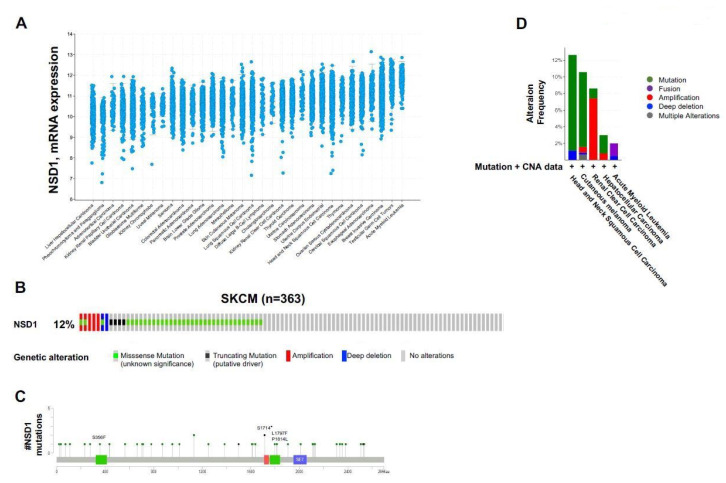
NSD1 expression and alterations in cancers. (**A**) NSD1 mRNA expression across TCGA tumor types. (**B**) Alterations in NSD1 gene are found in skin cutaneous melanoma (SKCM) (*n* = 363). Each sample is represented as a column. The image included all the NSD1 alterations found in the cohort and was cut down to fit the figure. (**C**) Lollipop mutation diagram mapper displaying NSD1 mutations in the SKCM cohort. Lollipop representations indicate the location of 4 nonsense and 39 missense mutations affecting NSD1. The lollipops are colored with respect to the corresponding mutation types. Truncating mutations are in black circles, missense mutations are in green circles. NSD1 functional domains with their respective mutations are indicated: PWWP domains are in green, SET domain is in blue and PHD finger is in red. Same height lollipops above the line indicate single NSD1 mutation per position, whereas the higher lollipops above the line indicate 2 mutations at a single position. These data were extracted from the SKCM TCGA PanCancer Atlas 2018 cohort of 363 patients. These figures were adapted from cBioPortal.org. (**D**) Alteration frequency of *NSD1* mutations and copy number alterations data in Skin cutaneous melanoma compared to Head and neck squamous cell carcinoma, renal clear cell carcinoma, hepatocellular carcinoma and acute myeloid leukemia. Alterations are distinguished with specific colors: missense mutations are in green, amplifications in red, deep deletions in blue, fusions in purple and multiple alterations in gray. All studies were selected from the TCGA PanCancer Atlas on cBioportal.org.

While *NSD1* expression varies across tumor types (Figure 2A), its enzymatic activity might also differ across tumor types.

NSD1 exerts tumor suppressive or promoter functions depending on the cellular context. Increased NSD1-SET activity has been implicated particularly in hematological malignancies, whereas loss-of function mutation or impaired expression characterize a wide variety of mostly solid human cancers [50] (Table S1).

*NSD1′s* somatic dysregulation, which results from transcriptional silencing associated with CpG island-promoter hypermethylation and translates into reduced H3K36 methylation, has been described in a variety of human cancers [51,52].

#### 2.3.1. Anti-Tumoral Role of NSD1

NSD1′s loss has been reported to be involved in a rare form of acute myeloid leukemia called acute erythroleukemia, which is generally associated with poor outcome [53]. Based on recent work showing that H3K36 methylation is crucial for erythroid differentiation [54], Leonards and colleagues have built a mouse model harboring hematopoietic-specific invalidation of Nsd1, that reveals impaired erythroblast differentiation and erythroleukemia induction [53]. Despite abundant expression of GATA1, the transcriptional master regulator of erythropoiesis, an impaired activation of GATA1-induced targets was observed. Thus, NSD1 functions as a co-regulator of GATA1-controlled erythroid differentiation and leukemogenesis most likely through the association with the transcriptional co-repressor SKI [53].

*NSD1* mutations have been associated with carcinoma of the upper airway digestive tract [52].

Furthermore, inactivating mutations in NSD1 in HNSCC and both inactivating mutations and deletions in *NSD1* in LUSC were reported. In HNSCC and LUSC, NSD1 loss of function is associated with DNA hypomethylation [20,55].

In HPV-negative HNSCC, NSD1 in vitro suppression led to the reduction of intergenic H3K36me2 domains followed by DNA hypomethylation, gain in H3K27me3 and, loss of the active mark H3K27ac, thereby affecting transcriptional activity. The aforementioned epigenome reprogramming led to the downregulation of putative genes associated with tumor immunity, signaling and plasticity [56,57].

Additionally, analysis of xenograft formed with NSD1-knocked down LUSC cells revealed immune cell exclusion within the tumor microenvironment compared to the wild-type LUSC cells [55]. Thus, in vivo *Nsd1* ablation triggers an immune cold phenotype that may favor tumor development and represent a marker of immune response. In HPV-positive HNSCC, expression levels of NSD members were not associated with change in lymphocyte infiltration but NSD low expression correlated with significantly reduced overall patient survival compared to HPV negative HNSCC [58].

Nevertheless, HPV-negative HNSCC with NSD1 mutations display better treatment responses to platinum-based chemotherapy compared to those lacking these mutations [59]. Of note, HPV-negative HNSCC with NSD1 mutations were associated with a decreased expression of *ERCC5* mRNA levels [59], and inactivation of ERCC5 has been previously shown to increase sensitivity to cisplatin in a variety of cancers [60,61]. Further investigation is required to link the improved response to platinum-based chemotherapy in HPV negative HNSCC NSD1 mutants to *ERCC5* decreased expression [59]. Another study in HPV negative-HNSCC patients stratified by their smoking history indicated that the frequency of *NSD1* mutations in HPV-negative HSNCC heavy smokers was significantly greater than never smokers and was associated with favorable prognosis [62]. The total mutational load was higher in NSD1-mutant tumors compared to *NSD1* wild-type tumors. Mutations in extra-cellular matrix (ECM) genes, including several collagen and laminin genes, were found in *NSD1*-mutant tumors [62]. Since these ECM component genes seem to be more altered, this could suggest that the ECM biochemical and biomechanical properties might also be altered which would impair tumor progression and metastasis formation, affecting then patients’ prognosis. Functional studies are required to assess the link between NSD1-mutant tumors and the ECM components in tumor aggressiveness. Another explanation is also related to immunogenicity as the higher mutational load detected in *NSD1*-mutant tumors may favor immune recognition and tumor elimination [62].

Furthermore, Su et al. showed in vitro that ccRCC samples harbored a pattern of DNA hypermethylation in NSD1′s promoter regions which correlated with NSD1′s silencing and with *SETD2* somatic mutations as well as high *EZH2* expression [63]. High *NSD1′s* hypermethylation level was observed in metastatic kidney tumors and often predicted advanced cancer stage and poor overall patient survival [63]. Moreover, high expression of EZH2 is reported to be significantly associated with advanced stage and is a predictor of aggressive tumor characteristics and frequent distant metastasis [64].

Alterations of *SETD2* have been also reported in ccRCC, and this might occur through a possible crosstalk between the inactivation of *NSD1* by methylation and *SETD2* [63].

Supporting the role of NSD1 loss in ccRCC tumorigenesis, Yan and colleagues found in RCC samples (TCGA cohort) a much higher level of NSD1 amplification in over 10% of ccRCC samples which was associated with a significant prolonged survival [65]. The mechanism by which NSD1 directly affects the survival rate of patients remains to be elucidated. Finally, ccRCC tumor samples harboring epigenetic silencing of *NSD1* displayed a specific genome-wide DNA methylation pattern consistent with the methylome signature observed in Sotos syndrome and HNSCC [20,55,63]. This methylome signature seemed to be associated with poor overall survival as compared to those without the methylome pattern [63]. Likewise, a NSD1 DNA hypomethylation signature that overlaps with the Sotos syndrome hypomethylation signature was observed in glioma and neuroblastoma [52].

Moreover, in altered H3K36 methylation settings, H3K27me3 would accumulate in regulatory elements of tumor suppressor genes due to EZH2-function, leading to their transcriptional repression, subsequently initiating poor cell differentiation, vascular invasion and tumor progression.

Epigenetic silencing of *NSD1*, through promoter hypermethylation, has been associated with neuroblastoma and gliomas. Moreover, in breast tumors, *NSD1* gene silencing was associated with reduced sensitivity to tamoxifen [66], thereby linking NSD1 loss to therapeutic resistance.

#### 2.3.2. Pro-Tumoral Role of NSD1

Somatic disruption of *NSD1* has been implicated in multiple tumor types, particularly hematological malignancies. The first mention of NSD1 role in tumorigenesis comes from the discovery in childhood acute myeloid leukemia (AML) of a translocation at t (5;11) (q35;p15.5) encoding a chimeric protein encompassing the carboxyl terminal of NSD1 that retains among other the catalytic SET domain fused to the FG-repeat domain of the nucleoporin protein NUP98, a component of the nuclear core complex. The NUP98-NSD1 fusion protein, reported in 5% of the cases, allows both H3K36 methyltransferase activity through NSD1 SET domain, and histone acetyltransferase activity via the NUP98 domain FG-repeats that interact with the histone acetyltransferase CBP/p300 [67,68]. Little is known about NSD1 target gene networks. Wang and coworkers proved in vivo that NUP98-NSD1 fusion protein enforces myeloid progenitor cells immortalization and blocks cellular differentiation through localization of H3K36 methylation and H3/H4 acetylation at promoter regions of stem-cell renewal oncogenes *Hox-A* and *Meis 1*, which subsequently leads to their transcriptional activation and contribute to leukemogenesis [68]. These data indicate that the concomitant presence of both active epigenetic marks, initiated by the NUP98-NSD1 fusion protein, is mandatory for *Hox-A* locus activation and AML induction. The epigenetic mechanism behind this activation could be insured by H3K36me blocking EZH2 complexes from binding to regulatory elements, hence preventing H3K27me3 mediated chromatin silencing.

Recently, Schmoellerl et al. identified CDK6 as a direct target of NUP98-NSD1 fusion protein in AML. CDK6 was found to be highly expressed in a novel mouse model allowing for regulatable expression of the NUP98-NSD1 signaling axis. CDK6 inhibition via a genetic or pharmacological approach using palbociclib led to a decreased leukemogenesis, with a rapid induction of apoptosis and cell cycle arrest [69].

Translocations involving NSD1 have been also detected in breast cancer [70].

In human HCC, NSD1 expression is higher in tumor tissues relative to normal tissue and its expression is associated with a reduced overall survival [71]. The authors showed in vitro that NSD1 enhanced HCC cell line proliferation, migration and invasion abilities, while the opposite was observed following NSD1 inhibition through regulation of WNT10B and the Wnt/β-catenin signaling pathway [71]. Mechanistically, NSD1 promotes Wnt10b transcription by inhibiting H3K27me3 methylation in the Wnt10b promoter region. H3K36me2/3′s accumulation following NSD1′s overexpression might create an imbalance with EZH2-H3K27me3′s distribution. As indicated previously, H3K27me3 and H3K36me3 are mutually exclusive on the same histone tail. Hence, disruptions in this cross-talk result in aberrant H3K27/H3K36 methylation motives and altered transcriptional profiles. Therefore, NSD1 expression represents a prognosis marker in HCC, and together with WNT10B, valid therapeutic targets.

In the same vein, in human colon cancer cell line HCT116, NSD1 has been reported to bind to the promoter proximal regions of a panel of genes, some, such as bone morphogenetic protein 4, may mediate its role in cancer and Sotos syndrome. Mechanistically, NSD1 may interact with H3K36 and RNA polymerase II (RNAPII) to regulate transcription and promote gene expression by stimulating the RNAPII’s elongation process [16].

In laryngeal tumors, characterized by high recurrence and poor overall survival, NSD1/2 mutations are associated with better prognosis, which provide relevant molecular prognostic biomarkers for treatment stratification [72].

While NSD1 has a selective nucleosomal preference for histone 3, it has been demonstrated that NSD1 has a non-histone substrate selectivity as well. Indeed, NSD1 has the ability to activate the p65 subunit of NF-KB, which is a central coordinator for immune and inflammatory responses, via the methylation of lysine 218 and 221 [73].

In conclusion, depending on the cellular context, and the chromatin modifying alterations, NSD1 may function then, as either an oncogene or as a tumor suppressor [23]. This could serve as an important element to take highly in consideration for the selection of patient therapy.

## 3. NSD1 in Melanoma

Cutaneous malignant melanoma is the deadliest form of skin cancer that originates from melanocytic cells. Although major breakthroughs were realized in the treatment of advanced melanomas these last decades, the responses are either transient or limited to restricted subsets of patients due to intrinsic or acquired resistance [74]. Several lines of evidence indicate that epigenetic mechanisms play a critical role in melanoma progression and response to treatment [2].

Skin cutaneous melanoma (SKCM TCGA dataset) showed *NSD1* alterations in 12% of the cases (*n* = 43/363). The highest frequency of alterations was attributed to missense mutations (*n* = 32), most of them occurring prior to the NSD1 SET catalytic domain. The other alterations included amplifications (*n* = 3), truncating mutations (*n* = 4), homozygous gene deletion (*n* = 1) (deep deletion) (Figure 2B).

As seen on the lollipop diagram mapper (Figure 2C), multiple NSD1 domains harbor point mutations. Indeed, one truncating mutation S1714 * was located on the PHD finger domain of two different patients. One missense mutation P2011S was identified on the catalytic SET domain of NSD1 responsible for its methyltransferase activity, and three missense mutations were located on the PWWP domains of NSD1, S356F, L1797F and P1814L. The point mutations affecting *NSD1*, specifically the ones located on the SET domain, might result in an inactive truncated NSD1 protein unable to perform its methyltransferase activity, which might create an epigenetic regulatory imbalance. Cutaneous melanoma has a very high prevalence of *NSD1* somatic mutations comparable to other cancer types that were discussed previously in this review (Figure 2D).

**Figure 3:**
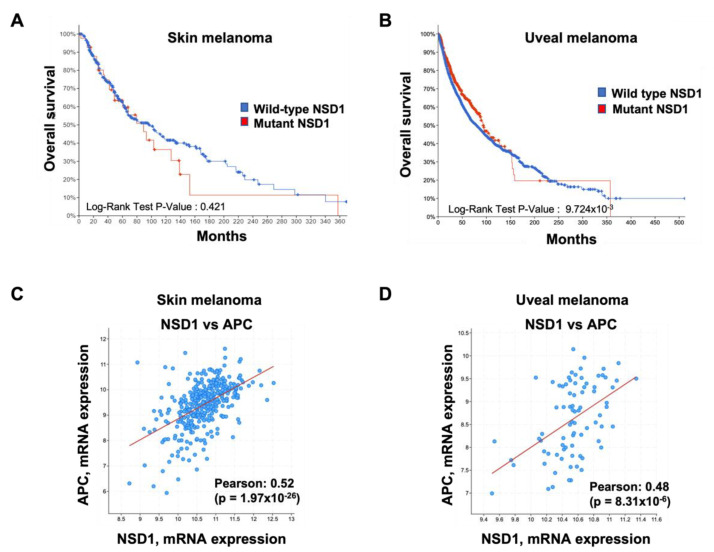
NSD1 mRNA expression in Uveal melanoma vs. Skin melanoma. (**A**,**B**) Kaplan–Meier curves showing overall survival stratified by *NSD1* expression in (**A**) skin melanomas and (**B**) uveal melanomas (SKCM and UM-TCGA, respectively, cBioPortal.org). The log-rank test was used for statistical analysis. (**C**) Scatterplot showing *NSD1* and *APC* mRNA expressions obtained from a skin melanoma cohort (cBioPortal.org). (**D**) Scatterplot showing *NSD1* and *APC* mRNA expressions obtained from a uveal melanoma cohort (cBioPortal.org).

As discussed earlier, alterations in *NSD1′s* expression have already shown promise as a prognostic biomarker in various cancers. However, Kaplan–Meier overall survival curves showed no statistically significant difference between patients with high and low NSD1 expression in either skin melanomas (Figure 3A) or uveal melanoma a rarer type of melanoma [75,76,77] which also presents with 1.3% of missense mutations (Figure 3B). While these analyses suggest that NSD1 expression does not influence overall survival in melanoma, given that NSD1 is an enzyme, one cannot rule out a correlation to activity than mRNA expression.

In the context of melanoma, NSD1′s role appears complex. Indeed, enhanced *NSD1* mRNA level was observed in cutaneous metastatic melanoma cells compared to skin melanocytes, suggesting that NSD1 is pro-tumoral. By contrast, a down-regulation of *NSD1* expression was observed during the progression from nonmetastatic to metastatic melanoma [78]. This discrepancy might be explained by the fact that melanoma is plastic and can switch between different phenotypes such as proliferative and invasive [79,80,81,82].

NSD1 expression may fluctuate to enable melanoma development and progression.

Further studies are most required to elucidate the mechanism by which *NSD1* acts in the pathogenesis of melanoma. To shed some light into a potential significant biological relevance of NSD1 in the pathogenesis of melanoma, we focused on co-expressed genes, highlighting the tumor suppressor APC as one of the top *NSD1* co-expressed genes (cBioPortal.org) (Figure 3C). NSD1 also highly correlates with APC in uveal melanoma (Figure 3B).

APC caught our intention given the implication of APC2-related member in Sotos syndrome [40] and its role as a negative regulator of the Wnt/β-catenin signaling pathway, which plays a critical role in cutaneous melanoma [83,84]. APC harbored a high frequency of methylation in primary and lymphatic cutaneous melanomas which was associated with transcriptional silencing [85]. APC loss of function may contribute to the upregulation of the Wnt/β-catenin signaling pathway, favoring melanoma progression [86].

By analogy with Sotos syndrome, we propose that in malignant melanomas, APC operates downstream NSD1. When NSD1′s activity is altered, the H3K36 active mark will not accumulate on *APC’s* promoter region; therefore the expression of the tumor suppressor *APC* remains repressed, subsequently the Wnt/β-catenin pathway will be constantly active, which would favor melanoma progression.

In the pathogenesis of melanoma, another epigenetic component known as EZH*2* seems to be highly involved. EZH2′s high expression is associated with aggressive tumor subgroups [87,88], and poor prognosis [87,89].

EZH2′s repressive epigenetic mark H3K27me3 is highly expressed in metastatic melanomas in comparison to primary melanomas [90]. The same group reported that H3K27me3′s expression in melanoma was highly correlated with tumor thickness and may represent a good marker to analyze EZH2′s activity as well as the PRC2 complex subunits. These data indicate that H3K27me3′s expression has a crucial role in melanoma dissemination, and it could be used as a suitable method to assess EZH2 inhibitor activity in clinical trials.

**Figure 4:**
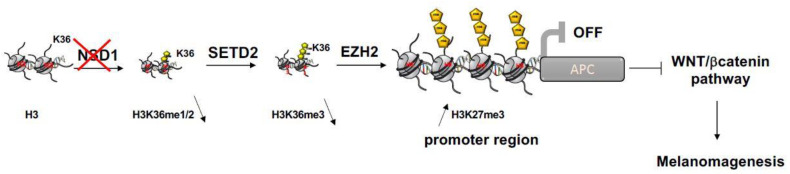
Epigenetic mechanisms driven by the histone lysine methyltransferases NSD1, SETD2 and EZH2 in cutaneous melanoma progression through WNT/bcatenin pathway regulation.

Taking these abovementioned data into consideration, we suggest a mechanism involved in melanoma by which *NSD1′s* loss-of-function is associated with a reduced epigenetic activating mark H3K36me1/2, therefore creating an epigenetic imbalance in favor with the accumulation of H3K27me3 on promoter regions of APC, leading to Wnt/β-catenin pathway regulation and subsequently to melanoma progression and immune resistance (Figure 4).

On the verge of these elements, a more thorough study is required since the mechanism depicting NSD1′s involvement in melanoma still has not been proven and is poorly understood.

## 4. Therapeutic Opportunities in the NSD1 Signaling Pathway

Considering their crucial role in the epigenetic regulation of cancer processes, selective inhibition of the histone methyltransferases NSD1, SETD2 and EZH2 could offer beneficial therapeutic strategies in treating cancer. Structural studies have shown that the catalytic SET domains of histone modifying enzymes play important roles conferring the intrinsic properties of these enzymes as well as their main activity. These studies could represent exploitable opportunities for designing specific inhibitors targeting the lysine methyltransferases [8,91].

Up until now, histone methyltransferase inhibitors have been rare, while selective inhibitors are currently under investigation [92]. The selective inhibition strategies could include blocking the methyl-donor in methyltransferase reactions or by blocking the binding of a specific substrate to the respective binding site of the protein methyltransferase [93].

Morishita et al., 2017 identified BIX-01294 as an NSD inhibitor [92]. BIX-01294 was primarily designed to inhibit H3K9 methyltransferases G9a and the related molecule GLP [94]. There are sequence similarity and structure conservation between catalytic SET domains of the NSD and the G9a/GLP proteins [95], and BIX-01294 demonstrated an in vitro inhibitory activity on all NSD proteins (NSD1, NSD2, NSD3) and H3K36me1 transcriptional mark.

Even though this compound does not distinguish the three members of the NSD family, these findings could benefit exploring specificities regarding each NSD member, and help developing selective NSD inhibitors required in cancers which originate from alterations affecting the *NSD* family.

On another note, Graham and collaborators performed molecular dynamics simulations of the post-SET loop region of NSD1, showing the existence of an autoregulatory position, which prevents the binding of the histone peptide and the entrance of the lysine-binding channel to the active site [96]. This loop must go through conformational change to enable histone binding and modification [8,96]. Hence, the dynamic behavior and potential conformations of NSD1 without or with the histone bound should be taken into account to design NSD1-targeting small molecules [96,97].

Based on these data, Huang et al. recently developed a first-in-class irreversible small molecule inhibitors of the NSD set domain family, BT5 molecule, with a distinct preference towards NSD1. BT5, which triggered downregulation of H3K36 epigenetic mark and associated target genes, impaired colony formation ability in NUP98-NSD1 leukemia cells [97]. One might also consider NSD1′s downstream effectors to target its NSD1 effects. For instance, given that the high expression of CDK6 was found in AML patients samples [69], palbociclib emerges as a rational therapeutic strategy for AML NUP98-NSD1 patients with poor prognosis. Other avenues in cancers where NSD1 displays a protumoral role include proteolysis targeting chimeras (PROTAC) technology, which utilizes the ubiquitin-protease system to target a specific protein, here NSD1, and induce its cellular degradation [98].

Downstream NSD1, EZH2 represents another pertinent druggable target. EZH2 inhibition using a selective chemical inhibitor GSK503, which targets the enzymatic activity of EZH2, significantly reduced the accumulation of H3K27me3 at tumor suppressor gene promoter regions, which lead to a significant decrease in tumor cells proliferation [89]. In vivo, studies using GSK503 on melanoma bearing-mice showed a significant reduction in tumor growth and invasive capacity thus preventing metastatic disease, which resulted in increased survival [89].

In the clinic, melanoma patients who were treated with immune checkpoints inhibitors targeting PD-1 (Programmed cell Death protein 1), PD-L1 (Programmed cell Death protein ligand 1) and CTLA-4 (Cytotoxic T-Lymphocyte-associated antigen 4) can become resistant and harbored an upregulated EZH2.

The clinical use of EZH2 inhibitor, GSK503, controlled melanoma growth, restored tumor immunogenicity among these patients, and reversed resistance to tumor immunotherapy [99]. Hence, it is suggested that targeting EZH2, with a highly selective inhibitor as seen above, alongside with immunotherapy could represent a good combinatory therapeutic strategy to tackle melanoma.

Another selective small molecule inhibitor of EZH2 methyltransferase activity, GSK126 acts on H3K27me3 global reduction and reactivates critical target genes that were previously repressed [100]. A reduced clonogenicity and stemness ability and metastasis were observed in uveal melanoma cells and patient-derived xenografts after treatment with GSK126 [101]. Nonetheless, clinical trials conducted with GSK126 on subjects with relapsed/refractory diffuse large B cell lymphoma, transformed follicular lymphoma, other non-Hodgkin’s lymphomas, solid tumors and multiple myeloma, were discontinued due to an unfavorable benefit risk profile (ClinicalTrials.gov identifier: NCT02082977).

More recently, a selective small molecule inhibitor of EZH2 “Tazemetostat (EPZ-6438)” has been clinically tested in patients with relapsed or refractory solid tumors, non-Hodgkin’s lymphoma, or histiocytic disorders with EZH2 and other gene mutations. The preliminary results from the phase II clinical trial demonstrated that 90% of patients with follicular lymphoma harboring an EZH2 mutation showed an objective response. This compound is on the verge of FDA approval following promising clinical trial results [102].

Recently, NSD1 loss has been reported to cause resistance to EZH2 inhibition [103] indicating that both EZH2 and NSD1 mutational status/activity should be considered when using EZH2 inhibitors in clinic.

It is worth noting that apart from EZH2, a variety of downstream effectors of NSD1 has been reported in different cancers [104]. Inhibition of the NSD1 module might require combination therapy to inhibit its pro-tumoral effect.

## 5. Conclusions

NSD1 is *a* methyltransferase that controls H3K36 methylation, an epigenetic mark usually associated with transcriptional activation. In humans, germline alterations of *NSD1* potentially inducing loss-of-function of the NSD1 protein have been associated with a developmental syndrome called Sotos. Sotos syndrome is characterized by overgrowth in early childhood and cancer predisposition. How NSD1 is regulated and functions in cancers is largely unknown.

Somatic dysregulation of *NSD1* has been associated with tumorigenesis. Depending on the cellular context, NSD1 exerts tumor suppressive or promoter effects. Further studies are needed to get insight into the molecular mechanism underlying its effects. Thus, in specific settings targeting NSD1 or its downstream effectors may be a potential strategy for tumor therapy.

## Supplementary Materials

The following supporting information can be downloaded at: https://www.mdpi.com/article/10.3390/cancers14194865/s1, Table S1: Effect of NSD1 across cancer types (promotes tumor growth/restricts tumor growth) and the type of mutation.
