# Supplementary material for: Lysine Methyltransferase NSD1 and Cancers: Any Role in Melanoma?
Source: Cancers (Basel). 2022 Oct 5;14(19):4865. doi: 10.3390/cancers14194865 (PMC9563040; doi:10.3390/cancers14194865)
Supplement: Supplementary file 1 [file cancers-14-04865-s001.zip › cancers-1920416-supplementary.pdf]

**Table S1.** Effect of NSD1 across cancer types (promotes tumor growth/restricts tumor growth) and the type of mutation.

| Cancer type | Alteration type | NSD1's role | References |
|---|---|---|---|
| ✓ Neuroblastoma & glioma | NSD1 gene inactivation via NSD1 promoter CpG island hypermethylation | Anti-tumoral | 53 |
| ✓ HPV-negative head and neck squamous cell carcinomas (HNSCC) | Several NSD1 Loss of function mutations were described : Missense, nonesense, frame_shift_del, frameshift insertion and splice mutations | Anti-tumoral | 57, 58, 59, 60, 61,62, 63, 64 |
| ✓ Lung squamous cell carcinoma (LUSC) | Inactivating point mutations were described, as well as NSD1 deletions | Anti-tumoral | 56 |
| ✓ Clear cell renal cell carcinoma (ccRCC) | DNA hypermethylation on NSD1 promoter regions | Anti-tumoral | 65, 66, 67 |
| ✓ Hepatocellular carcinomas (HCC) | NSD1 overexpression | Pro-tumoral | 73 |
| ✓ Laryngeal tumors | Several NSD1/2 inactivating mutations were described : Missense, nonesense, frame_shift_del, frameshift insertion, mutations | Pro-tumoral | 74 |
| ✓ Acute Myeloid Leukemia (AML) | t(5;11)(q35;p15.5) Translocation fuses NSD1 to NUP98 | Pro-tumoal | 69, 70, 71 |
| ✓ Breast cancer | NSD1 translocations | Pro-tumoral | 72 |
